# Significance of Direct and Indirect Impacts of Temperature Increase Driven by Climate Change on Threat to Oilseed Rape Posed by *Sclerotinia sclerotiorum*

**DOI:** 10.3390/pathogens12111279

**Published:** 2023-10-25

**Authors:** Marek Wójtowicz, Andrzej Wójtowicz

**Affiliations:** 1 Plant Breeding and Acclimatization Institute—National Research Institute in Radzików, 60-479 Poznan, Poland; m.wojtowicz@ihar.edu.pl; 2 Institute of Plant Protection—National Research Institute, 60-318 Poznan, Poland

**Keywords:** oilseed rape, Sclerotinia stem rot, climate change

## Abstract

Sclerotinia stem rot, caused by *Sclerotinia sclerotiorum*, threatens oilseed rape (*Brassica napus*) crops internationally. The development of this disease is strongly controlled by the weather, which is why global climate change is likely to influence its spread and impact. Climate change may affect the pathogen directly or indirectly via the host plant. This study investigates the potential impact of climate warming on Sclerotinia stem rot severity in oilseed rape in Poland. The aim of this investigation was to assess the relationship between the direct impact (DI) and indirect impact (II) of climate change on disease severity using the 4.5 and 8.5 representative concentration pathways (RCPs). Under the RCP4.5 scenario, nearly 60% of the simulations performed for 16 regions in four periods (2020–2039, 2040–2059, 2060–2079, 2080–2099) showed reductions in disease severity in comparison to those conducted for 1986–2005, while under RCP 8.5, this reduction was generated for nearly 90% of the cases. The effect of the RCP scenario on clustering the regions according to the value of Sclerotinia stem rot severity was also investigated. The simulations revealed that, for all periods, the lowest disease severities are expected for Zachodniopomorskie and Pomorskie. The results obtained also show the superior effects of the II over the DI on Sclerotinia stem rot severity in the future. Under the RCP4.5 scenario, the rate of IIs was greater than that of DIs for 10 regions, while under RCP8.5, this relationship was registered for 16 regions. These outcomes result from the acceleration of the oilseed rape flowering period triggered by expected temperature increases. The novelty of this study lies in a detailed analysis of the relationships between the DI and II of climate change, expressed numerically, for 16 regions in Poland. The obtained results highlight the role of the indirect impact in shaping disease severity and indicate that it should be incorporated into assessment methods of climate change effects alongside the direct impact.

## 1. Introduction

Oilseed rape is the third most important source of both vegetable oil (after soybean and oil palm) [1] and oil meal (after soybean and cotton) in the world [2]. Over the last 20 years, the worldwide production of this crop has doubled from 36,019,847 to 71,333,435 tons [3]. The main producers of oilseed rape are China (17% of the global output), Canada (16%), India (12%), Australia (5.5%), Germany (4.1%), France (3.8%), Poland (3.5%), Ukraine (3.4%), and the Russian Federation (3.2%). The differences between the highest and the lowest oilseed rape yields registered in the years 2017–2021 for the leading producers of this crop are as follows: 39% (Australia), 35% (Canada), 25% (Russian Federation), 22% (France), 21% (Ukraine), 19% (Poland and Germany), and 14% (India). The principal reasons for variability in the crop yield, besides the yield potential of a cultivar, are the meteorological conditions and pests [4,5]. 

One of the most destructive and harmful diseases of oilseed rape is Sclerotinia stem rot, which is caused by *Sclerotinia sclerotiorum* [6]. Among the 10 most significant biotic threats to oilseed rape cultivation, this pathogen holds first place in China and second in Australia, Europe, and Canada [7]. 

Yield losses due to Sclerotinia stem rot have been reported to be as high as 80% in China [8] and 75% in Nepal [9]. In the United Kingdom, oilseed rape infestation by *S. sclerotiorum* results in a 50% yield loss [10], while in Germany, it results in a 20–30% yield loss [11]. In Saskatchewan, Canada, and in Minnesota and North Dakota, USA, in 1991–1997, yield losses due to Sclerotinia stem rot ranged from 11.4 to 14.9%, ref. [12], 11.2 to 13.2%, and 5 to 13% [13], respectively. 

Also, in Poland, along with Phoma lingam and clubroot, Sclerotinia stem rot is considered to be among the most dangerous oilseed rape diseases [14]. According to Starzycki and Starzycka, this disease occurs in Poland every year to a greater or lesser extent [15]. In an experiment conducted in the years of 2005–2009 focusing on oilseed rape infestation by *S. sclerotiorum*, Jajor et al. registered 3–27% of the surveyed plants as having symptoms of the disease [16]. Great diversity in the number of oilseed rape plants infested with *S. sclerotiorum* was found by Kaczmarek et al. and Bracharczyk et al., who, in the years of 2010–2011, observed that 2–34% and 25–50% of plants, respectively, had Sclerotinia stem rot [17,18]. The importance of this disease is even better characterized by the results of research on its impact on the yield of oilseed rape. In a study on the optimization of the dates of fungicidal treatments against Sclerotinia stem rot in oilseed rape, Bracharczyk et al. [18] recorded yield losses of 6% and 19%, depending on the year. High yield losses of oilseed rape caused by *S. sclerotiorum* were also registered by Jajor et al. [16]. While examining the impacts of fungicide protection and meteorological conditions on the infection of oilseed rape cultivars by *S. sclerotiorum*, the authors observed yield losses of up to 15%. According to Starzycki and Starzycka, yield losses may even exceed 30% under conditions that are extremely favorable for the development of the pathogen [15]. 

*S. sclerotiorum* survives in the soil as sclerotia from previous epidemics [19]. Sclerotia germinate either myceliogenically, by producing mycelia, or carpogenically, by producing apothecia, which, in turn, release ascospores [20]. The infection of oilseed rape leaves by *S. sclerotiorum* occurs primarily as a result of ascospore germination on petals, through which the mycelium of the pathogen penetrates into the leaf tissue [21,22]. When ascospores come into direct contact with leaf tissue, they either do not germinate [22] or their ability to germinate is strongly reduced [23]. A strong correlation between petal infestation and disease incidence explains why the flowering period of oilseed rape is critical for the epidemiology of Sclerotinia stem rot [22,24]. 

The development of this disease is also affected by meteorological factors, among which air temperature, in addition to air humidity, is commonly considered to be a major determinant of Sclerotinia stem rot risk [25,26,27]. Knowledge about the relationship between temperature and pathogen development is widely used to predict the disease threat to crops as a result of climate change [28,29,30]. 

According to the Synthesis Report of the IPCC, in the first two decades of the 21st century, the global surface temperature was 0.99 °C higher than in 1850–1900, and it has increased faster since 1970 than in any other 50-year period over at least the last 2000 years [31]. Depending on the scenario, in 2081–2100, the Earth’s surface temperature is very likely to have increased by an average of 1.4 °C to 4.4 °C compared to that in 1850–1900. 

Such changes in temperature could profoundly alter pathogen threats to crops in the future [32,33,34]. The status of crop diseases may be affected by climate change directly or indirectly [35]. The direct impact of climate change results from the immediate reactions of living organisms to changed meteorological conditions, while the indirect results come from changes in the compositions, structures, and functions of ecosystems triggered by changes in meteorological variables [36]. 

In the present article, a direct impact (DI) is a relationship between temperature and *S. sclerotiorum* development, while an indirect impact (II) represents the influence of temperature on the development of oilseed rape—the *S. sclerotiorum’s* host crop. The complex nature of the influence of climate change on crop disease development makes it difficult to precisely define relationships between DIs and IIs. For this reason, despite the general agreement on the distinction between these two methods of climate change impacts, it is not easy to find articles that deal with plant disease in which the relationships between the DI and II are expressed in numbers. Among the crops most often taken into account, in the last twenty years, the studies describing pathogen responses to climate change on the basis of simulation results have mostly included wheat, followed by rice, grapevine, and potato, whereas oilseed rape is under-represented [37]. 

The present work intends to fill the gap in information about the relationships between *S. sclerotiorum* and oilseed rape development under climate change conditions. We are especially interested in the relationships between the DI and II of climate change at the level of Sclerotinia stem rot risk. Our earlier studies indicated the superiority of the II over the DI in determining the fatty acid composition of oilseed rape oil on a national scale. We hypothesized that II may also be dominant in shaping the future Sclerotinia stem rot threat to oilseed rape. The objective of the present study was to check this hypothesis by comparing the DI and II of climate change on the level of Sclerotinia stem rot risk. We express the relationships between these phenomena numerically on the regional scale. The regions of Poland that are expected to experience major increases in *S. sclerotiorum* pressure on oilseed rape in the future are also indicated. 

## 2. Materials and Methods

### 2.1. Development of a Model for Simulating the Flowering Time of Oilseed Rape 

Our model was developed on the basis of data collected in field experiments conducted at Winna Góra (60 km south of Poznań) in 2005–2022. Each year, three oilseed rape cultivars were used in the study (Table 1). The data gathered in odd years were used to build the model, while model validation was performed on data from even years.

The model development consisted of quantifying the relationship between the accumulated rapeseed developmental rate (ARDR) and the phenological development stages of the cultivars listed in Table 1, according to the BBCH scale [38]. The phenological development stages were registered twice per week in the period from the first of January to the end of oilseed rape flowering period each year. Detailed information explaining how to calculate the rapeseed developmental rate (RDR) was presented by Racca et al. [39]. The collected data were used to build a model simulating oilseed rape development. The model consisted of two submodels (Table 2). The first one describes oilseed rape development from 1 January to the beginning of flowering period (BBCH 61), while the second covers the period from the beginning to the end of flowering (BBCH 61–69) (Table 2). The validation of the model was based on a comparison of the simulated and observed results. 

### 2.2. Development of a Model for Estimating Sclerotinia Stem Rot Severity in Oilseed Rape at Flowering Time

The model was developed using the information collected by Koch and presented in her doctoral thesis [22]. The data shown in this study illustrate the disease severity registered at different temperatures as an effect of the infection of oilseed rape stalks by ascospores. To mathematically describe the pathogen response to the stimulus, we used two submodels. Both define the effect of the temperature upon disease severity. The first, double logistic, operates in the range of 6–18 °C, while the second, based on the Beta function, operates in the range of 18–26 °C (Table 3).

### 2.3. Projection of the Effect of Climate Change on the Sclerotinia Stem Rot Severity in Oilseed Rape 

**Figure 1:**
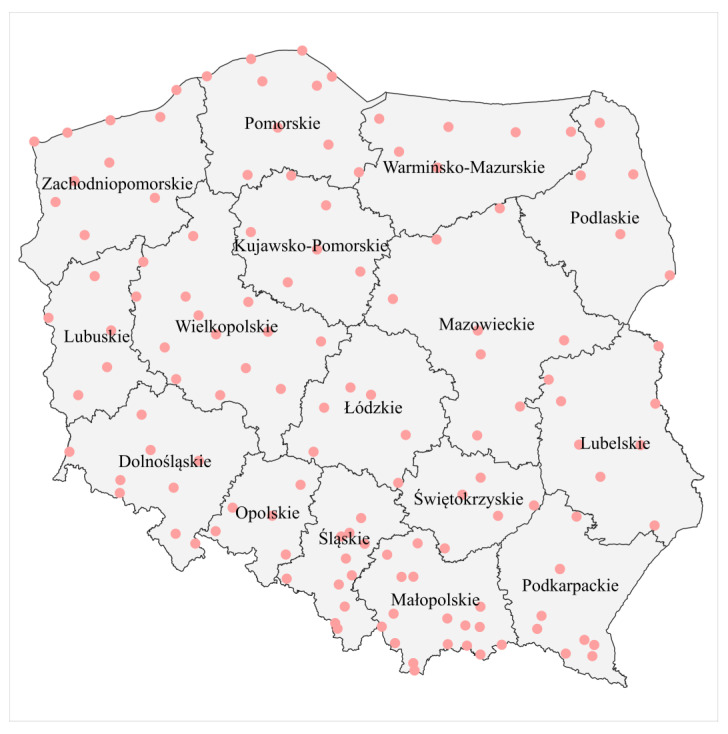
Spatial distribution of the 133 meteorological stations used in the study.

The simulations were performed using two sets of meteorological data. The first consisted of data registered in the period of 1986–2005 at 133 locations in Poland (Figure 1).

The second was obtained after transforming the recorded data to reflect temperature changes under the RCP4.5 and RCP8.5 scenarios according to the 29 climate models presented on the Climate Change Knowledge Portal created by the World Bank (https://climateknowledgeportal.worldbank.org/download-data (accessed on 15 January 2023) (Table 4).

The transformation of meteorological data was conducted using the delta change approach, which consists of adding the mean monthly change value for the model results for the projected and registered periods to an observed time series according to Equation (1):

Tdb = Tobsref + (Tsimeproj − Tsimref)(1)

where Tdb is the debiased temperature; Tobsref is the daily temperature observed in the reference period; Tsimproj is the monthly temperature simulated for the projected period; and Tsimref is the monthly temperature simulated for the reference period.

The next task of the study was to estimate the effect of climate change on Sclerotinia stem rot severity in oilseed rape. This was completed using simulations of the impact of temperature on both pathogen development and the oilseed rape flowering period. This task was accomplished in three steps. The first step was to determine the dates of the beginning and the end of the oilseed rape flowering period (BBCH 61, BBCH 69). In the second step, the disease severity for each day of the flowering period was calculated. The third step consisted of calculating the sum of disease severity in the flowering period for each region (Dolnośląskie, Kujawsko-Pomorskie, Lubelskie, Lubuskie, Łódzkie, Małopolskie, Mazowieckie, Opolskie, Podkarpackie, Podlaskie, Pomorskie, Śląskie, Świętokrzyskie, Warmińsko-Mazurskie, Wielkopolskie, Zachodniopomorskie), scenario (RCP4.5, RCP8.5) and period (2020–2039, 2040–2059, 2060–2079, 2080–2099).

Subsequently, we grouped the regions according to disease severity by applying a cluster analysis using the Euclidean distance in such a way that the connection level within the same group was as high as possible, whereas within combinations of other groups, it was as small as possible. Distinct groups of regions depending on disease severity were formed on the basis of the Euclidean distance equaling 1.0. 

The next part of the study was designed to measure the impact of climate change on disease severity. To fulfill this task, in addition to the simulation results collected so far, information about disease severity for both the registered (1986–2005) and projected periods (2020–2039, 2040–2059, 2060–2079, 2080–2099) without the influence of temperature on oilseed rape flowering was needed. This is why another series of simulations was conducted. This time, the dates of oilseed rape flowering indicated for the period of 1986–2005 were also used in simulations for the other periods. On the basis of both simulation series, the impact of climate change on disease severity was calculated according to the equations presented below.

The direct impact of temperature on disease severity was calculated using Equation (2):

DI = ds − tor i, j − ds1986–2005(2)

where DI is the direct impact of climate change on disease severity; ds–tor i, j is the disease severity obtained from simulations without allowing for the influence of temperature on the development of oilseed rape for scenario i and period j (i: RCP4.5, RCP8.5; j: 2020–2039, 2040–2059, 2060–2079, 2080–2099); and ds1986–2005 is the disease severity obtained in simulations for the period of 1986–2005.

The sum of the direct and indirect impacts of climate change on disease severity was calculated using Equation (3):

(D + I)I = ds + tor i, j − ds1986–2005(3)

where (D + I)I is the sum of the direct and indirect impacts of climate change on disease severity; ds+tor i, j is the disease severity obtained in simulations allowing for the influence of temperature on the development of oilseed rape for scenario i and period j (i: RCP4.5, RCP8.5; j: 2020–2039, 2040–2059, 2060–2079, 2080–2099); and ds1986–2005 is the disease severity obtained in simulations for the period of 1986–2005.

The indirect impact of climate change on disease severity was calculated using Equation (4):

II = (D + I)I − DI(4)

where II is the indirect impact of climate change on disease severity; (D + I)I is the sum of the direct and indirect impacts of climate change on disease severity; and DI is the direct impact of climate change on disease severity.

For further analysis, the accumulated rate of change (AROC) of the direct impact (DI) and indirect impact (II) of climate change over time was calculated using Equation (5):

(5) $$AROC=\sum_{i=1}^{N}\left(I_{i+1}-I_{i}\right)/T$$

where AROC is the accumulated rate of change; Ii is the impact measured at period i; Ii + 1 is the impact measured at period i + 1; T is the time taken for that change to occur; and N is the total number of observations. 

## 3. Results

### 3.1. Development of a Model for Simulating the Flowering Time in Oilseed Rape

The submodels expressing the relationship between temperature and the oilseed rape flowering period are presented in Table 5.

**Figure 2:**
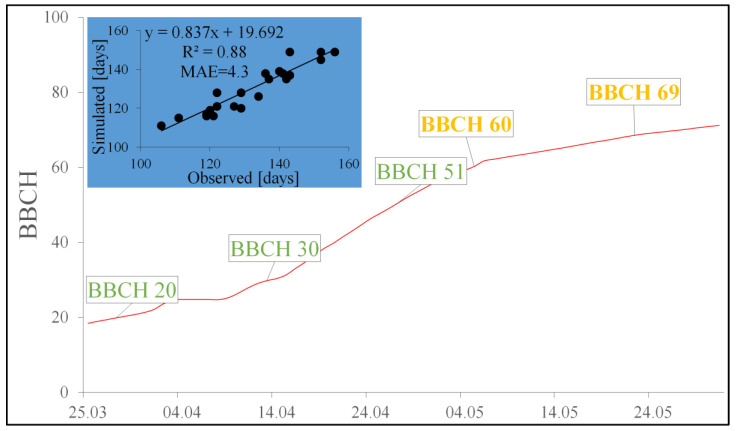
Simulation of the oilseed rape flowering period (indicated by a red curve) and the relationship between the observed and simulated flowering phenophases: BBCH 60, BBCH 69 in even years—2006, 2008, 2010, 2012, 2014, 2016, 2018, 2020, 2022 (represented in the chart by a blue background).

The comparison of the observed beginning of the oilseed rape flowering period and that simulated using the models developed revealed that the differences depending on the year did not exceed 9 days (Table 6). Even better results were obtained when we compared the observed and modeled ends of the flowering period. This time, the disagreement ranged between 1 and 7 days. The mean absolute error (MAE) obtained when we compared the simulated and observed start times of the flowering period, expressed in days of the year, was 4.6, while for the end of the flowering period, the MAE was 3.9. When the results of the beginning and end of the flowering period were put together, the differences between the observed and simulated results measured with the MAE was 4.3 (Figure 2), while the agreement between the observed and simulated results measured with R^2^ was 0.88 (Figure 2).

### 3.2. Development of a Model for Estimating Sclerotinia Stem Rot Severity in Oilseed Rape at Flowering Time

**Figure 3:**
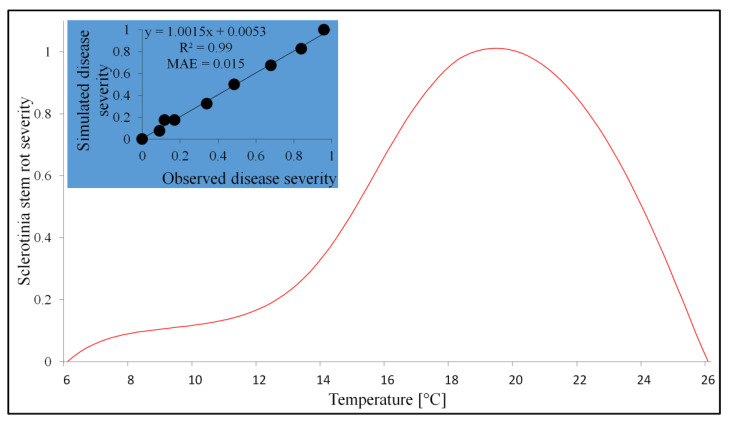
The effect of temperature on Sclerotinia stem rot severity (indicated by a red curve) and comparison of the observed and simulated values of that parameter (represented in the chart by a blue background).

The model expressing the relationships between temperature and Sclerotinia stem rot severity consists of two submodels. The first one, double logistic, estimates the disease severity triggered by temperatures in the range of 6–18 °C, while the second is based on Beta functions in the range of 18–26 °C (Table 7). The relationship between the data and the model output is presented in Figure 3. 

### 3.3. Projection of the Effect of Climate Change on Sclerotinia Stem Rot Severity in Oilseed Rape 

#### 3.3.1. Simulation of the Effect of Climate Change on the Oilseed Rape Flowering Period

The simulation based on meteorological data registered in the years of 1986–2005 generated average starts of the oilseed rape flowering stage, depending on the region analyzed, of between 122 (02.05 lubuskie) and 136 (16.05 małopolskie) days after 1st January (Table 8). For the RCP4.5 scenario, the simulation revealed reductions in the period from the 1st of January until the beginning of the flowering period of 8–13 (2020–2039), 11–17 (2040–2059), 15–22 (2060–2079), and 17–25 (2080–2099) days. The minimum and maximum reductions for the RCP8.5 scenario spanned between 8 and 13 (2020–2039), 16 and 23 (2040–2059), 25 and 35 (2060–2079), and 33 and 46 (2080–2099) days. 

The conducted simulations showed that, in the years of 1986–2005, the flowering period ended between 143 (26.05 lubuskie) and 157 (06.06 pomorskie) days after the 1st of January. In the simulations performed for the scenario of RCP4.5 the end of flowering was generated to occur 8–12 (2020–2039), 11–16 (2040–2059), 15–21 (2060–2079), and 16–23 (2080–2099) days earlier. For the RCP8.5 scenario, the reductions in the period between the first of January and the end of flowering were 8–12 (2020–2039), 15–21 (2040–2059), 23–32 (2060–2079), and 30–41 (2080–2099) days.

The calculation performed on the simulation results showed that, in the years of 1986–2005, the flowering period lasted 20–22 days. The lengths of that period obtained in simulations for the RCP4.5 scenario were 20–23 (2020–2039), 19–24 (2040–2059), 20–24 (2060–2079), and 20–25 (2080–2099), respectively, whereas for the RCP8.5 scenario, they ranged from 20 to 23 (2020–2039), 21 to 25 (2040–2059), 22 to 26 (2060–2079), and 23 to 27 (2080–2099).

#### 3.3.2. Simulation of the Effect of Climate Change on Sclerotinia Stem Rot Severity 

The simulations performed on the data collected in the years of 1986–2005 showed that Sclerotinia stem rot severity varied between 7.04 in Zachodniopomorskie and 9.16 in Podlaskie (Table 9). Under the RCP4.5 scenario, the minimum values of disease severity were generated for Zachodniopomorskie (6.97 (2020–2039), 6.72 (2060–2079), and 6.52 (2080–2099)) and Pomorskie (6,90 (2040–2059)). The maximum values were generated for Lubelskie (9.28 (2020–2039), 9.35 (2040–2059), 9.51 (2060–2079), and 9.44 (2080–2099)). For the RCP8.5 scenario, irrespective of the period, the minimum and maximum Sclerotinia stem rot severities were revealed in simulations for Zachodniopomorskie (respectively, 6.93, 6.39, 5.78, and 5.40) and Lubelskie (respectively, 9.23, 9.05, 8.96, and 8.42). The coefficient of variation (CV) of Sclerotinia stem rot severity obtained in the simulations conducted on meteorological data registered in 1986–2005 was 0.076 (Table 9). Similar results were generated for the period of 2020–2039 under the RCP4.5 (0.077) and RCP8.5 climate scenarios (0.078). Greater variation in disease severity between regions was registered for the other periods. Under RCP4.5, the coefficients of variation were 0.084, 0.097, and 0.103, while under RCP8.5, they were 0.097, 0.124, and 0.128, respectively, for 2040–2059, 2060–2079, and 2080–2099.

**Figure 4:**
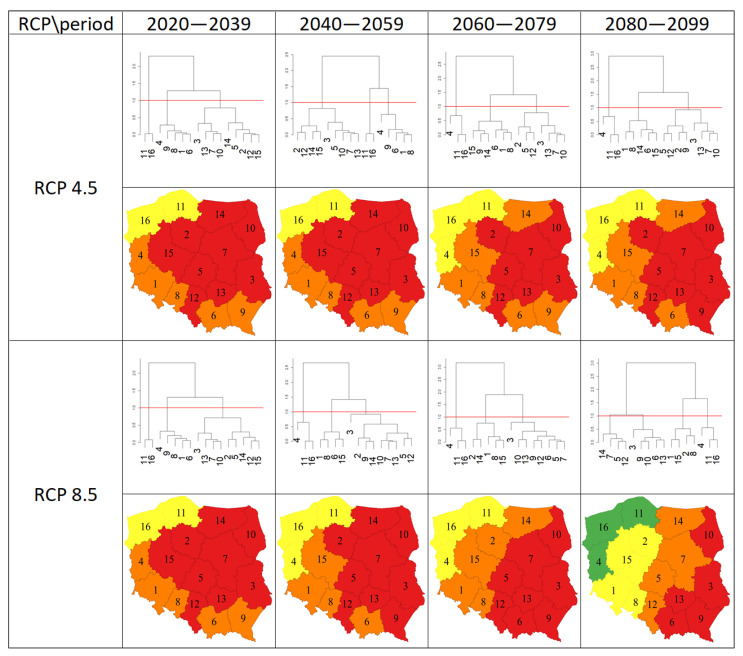
Results of clustering the regions according to the value of Sclerotinia stem rot severity for the two RCP scenarios (4.5, 8.5) and four periods (2020–2039, 2040–2059, 2060–2079, 2080–2099). Regions in the same cluster are marked with the same color. The numbers on the dendrograms and maps represent the regions arranged in alphabetical order (1—Dolnośląskie, 2—Kujawsko-Pomorskie, 3—Lubelskie, 4—Lubuskie, 5—Łódzkie, 6—Małopolskie, 7—Mazowieckie, 8—Opolskie, 9—Podkarpackie, 10—Podlaskie, 11—Pomorski, 12—Śląskie, 13—Świętokrzyskie, 14—Warmińsko-Mazurskie, 15—Wielkopolskie, 16—Zachodniopomorskie).

The results of clustering the regions on the basis of a Euclidean distance equal to 1.0 according to Sclerotinia stem rot severity are shown in Figure 4. Under the scenario of RCP4.5, irrespective of the period, the regions were grouped into three clusters. Three clusters were also distinguished under the RCP8.5 scenario for the periods of 2020–2039, 2040–2059, and 2060–2079, while for the period of 2080–2099, the regions were grouped into four clusters. Under the scenario of RCP4.5, the number of regions belonging to the cluster characterized by the smallest disease severity was two for 2020–2039 and 2040–2059, while it was three for the two other periods. The number of regions with the highest disease severity was nine for 2020–2039 and 2040–2059, while it was seven and eight, respectively, for 2060–2079 and 2080–2099. Under the RCP8.5 scenario, the number of regions with the smallest disease severity was two for 2020–2039, while it was three for the other periods. The number of regions characterized by the highest disease severity was nine for 2020–2039 and 2040–2059, while it was eight and five for 2060–2079 and 2080–2099, respectively. 

**Figure 5:**
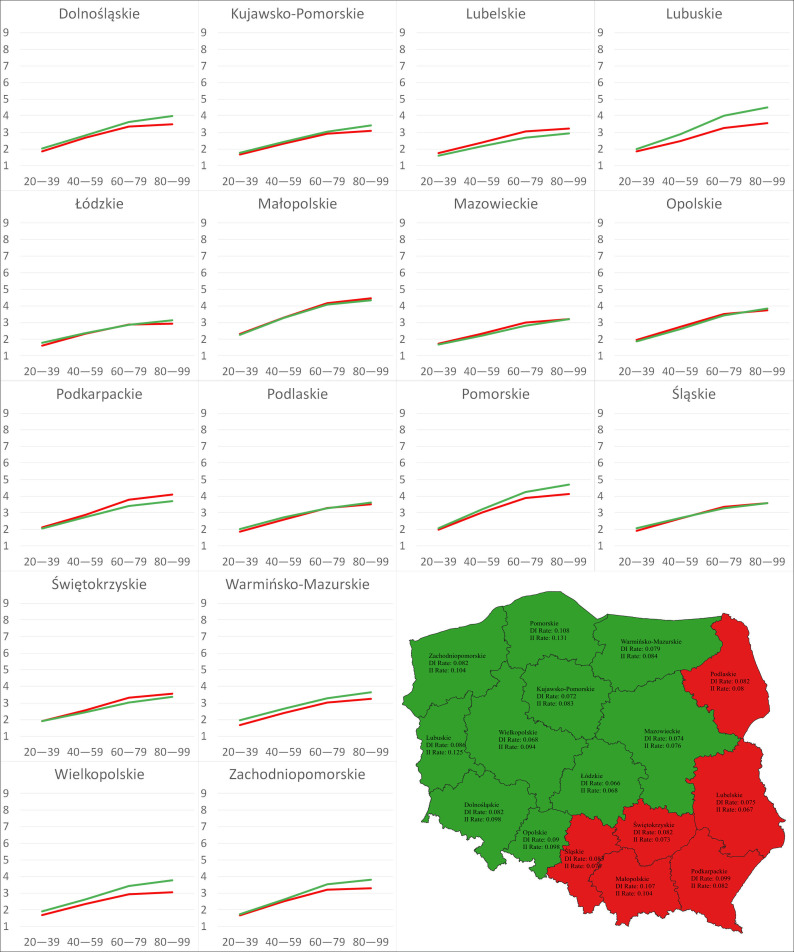
Charts: DI and II of climate change over time under the RCP4.5 scenario in 16 regions. Map: Distribution of the regions based on relationships between the AROCs of the DI and II on Sclerotinia stem rot severity under the RCP4.5 scenario. Red indicates AROC of DI > AROC of II; green indicates AROC of DI < AROC of II.

**Figure 6:**
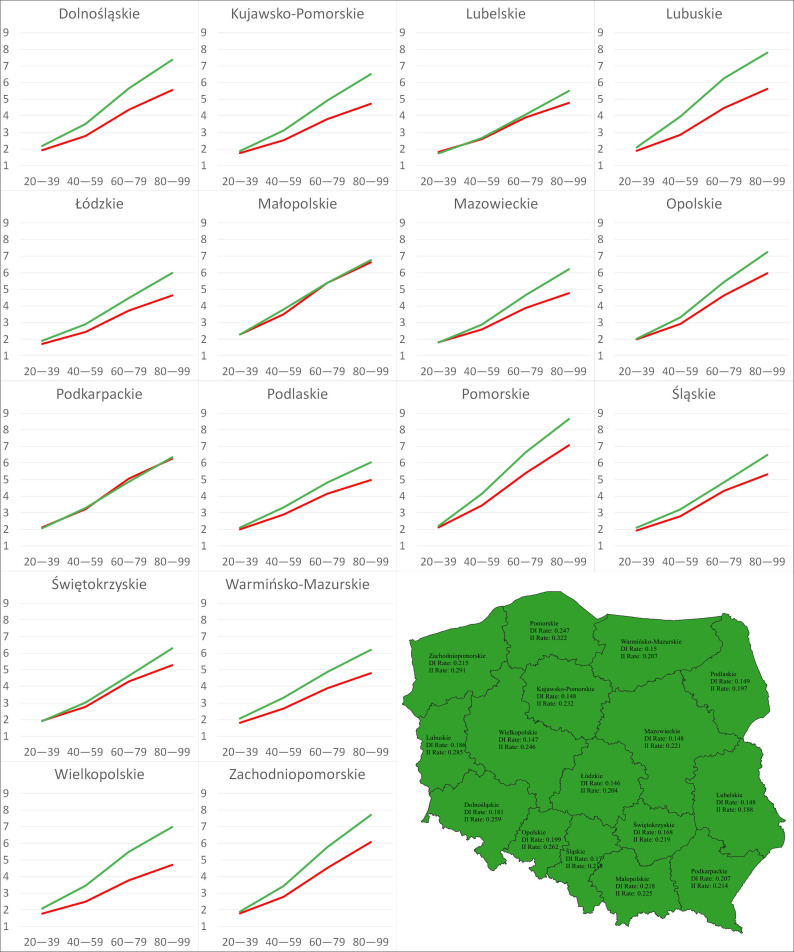
Charts: DI and II of climate change over time under the RCP85 scenario in 16 regions. Map: Distribution of regions based on relationships between the AROCs of the DI and II on Sclerotinia stem rot severity under the RCP8.5 scenario. Red indicates AROC of DI > AROC of II; green indicates AROC of DI < AROC of II.

The calculations showed systematic increases in the absolute values of both the DI and II of climate change on disease severity over time (Table 10). The minimum and maximum values of the DI under the RCP4.5 scenario were 1.62 in Łódzkie and 2.32 in Małopolskie, respectively, from 2020 to 2039, whereas they were 2.32 in Kujawsko-Pomorskie and 3.29 in Małopolskie, respectively, for 2040–2059 The analyzed parameter ranged between 2.88 in Łódzkie and 4.18 in Małopolskie for the period of 2060–2079 and 2.93 in Łódzkie and 4.46 in Małopolskie for 2080–2099. Under the RCP8.5 scenario, the calculation revealed that the minimum values of the DI were generated for Łódzkie (1.72 for 2020–2039, 2.44 for 2040–2059, 3.73 for 2060–2079, 4.64 for 2080–2099), irrespective of the period. The maximum values of that parameter were 2.28, 3.50, and 5.36 for Łódzkie for 2020–2039, 2040–2059, and 2060–2079, respectively, whereas the maximum value was 7.06 in Pomorskie for 2080–2099. The calculations that focused on the II of climate change under the RCP4.5 scenario revealed that the smallest values of that parameter were generated for Lubelskie: −1.60 (2020–2039), −2.17 (2040–2059), −2.68 (2060–2079), and −2.94 (2080–2099). The greatest values were −2.27 and −3.28 for Małopolskie and −4.25 and −4.70 for Pomorskie. Under the RCP8.5 scenario, the smallest II was generated for Lubelskie, with values of −1.74 (2020–2039), −2.67 (2040–2059), −4.08 (2060–2079), and −5.50 (2080–2099), while the greatest values were generated for Małopolskie (−2.27) and Pomorskie (−4.15, −6.64, −8.65). The accumulated rates of change (AROCs) for the DI and II over time are presented in Figure 5 and Figure 6.

Under the RCP4.5 scenario, the AROCs of the II were greater over time than those expressing the rate of change in the DI for 10 regions (Dolnośląskie, Kujawsko-Pomorskie, Lubuskie, Łódzkie, Mazowieckie, Opolskie, Pomorskie, Warmińsko-Mazurskie, Wielkopolskie, Zachodniopomorskie), whereas under the RCP8.5 scenario, the AROCs of the II were greater than the opposite ones for all regions. For both scenarios, the maximum values of the AROCs of the DI and II were registered for Pomorskie (RCP4.5: 0.108, 0.131; RCP8.5: 0.247, 0.322), while the minimum AROCs of the DI were found for Łódzkie (RCP4.5: 0.066, RCP8.5: 0.146) and the minimum AROCs of the II were found for Lubelskie (RCP4.5: 0.067, RCP8.5: 0.188)

## 4. Discussion

The present study focused on an exploration of the relationships between the direct impact and indirect impact of climate change on the level of Sclerotinia stem rot risk. On the basis of the meteorological data analyzed, we found that the flowering period of oilseed rape is expected to start and end earlier in the future, irrespective of the RCP scenario. In both scenarios, climate change affects the start date of the flowering period more than the end date. Moreover, these changes are dependent on the period. Over time, they will grow from the smallest for 2020–2039, reaching the highest values in 2080–2099. The simulation also revealed that the changes expected under the RCP8.5 scenario are greater than under RCP4.5 for all periods analyzed. 

These findings are in agreement with the study results presented by Hájková et al. [40], who noticed a significant shift in the beginning of the oilseed rape flowering period. The authors demonstrated that the onset of flowering advanced progressively in the Czech Republic, and the differences between the start of that phenophase in 1991 and 2012 reached almost 15 days. Further studies of earlier-flowering plants in response to ongoing climate change have been registered in other European countries [41,42]. For example, Parmesan and Yohe [43] confirmed this trend by analyzing the flowering periods of 461 plant species. According to Franks et al. [44], the shifts in phenophases are largely attributed to rising temperatures. In agreement with these findings, Hájková et al. [40] showed that the best predictor for the onset of oilseed rape flowering is the mean air temperature. The close relationship of oilseed rape flowering with this parameter was also demonstrated by Wójtowicz [45], who studied the effect of the mean air temperature registered in April on the start of that phenophase. The present study findings, which deal with the onset of flowering period, are also reflected by the results obtained in the studies performed with the use of models. Racca et al. [37] showed that the flowering of oilseed rape compared to the period of 1970–2000 is expected to start earlier, from 17–19 to 30–49 days for 2020–2055 and 2070–2100, respectively. 

Our study demonstrated that the oilseed rape flowering period is expected to be prolonged in the future, mainly due to an earlier start of that phenophase under warming conditions. Similar conclusions were reached by Mo et al. [46], who analyzed data including 136 plant species from 217 observational sites across eight climatic zones in China from 1963 to 2013 and stated that flowering duration was prolonged, mainly because the beginning of flowering was more substantially advanced than the end of the flowering period. 

The earlier start of the flowering period that was proven in the present study may implicate earlier infections of oilseed rape caused by *S. sclerotiuorum*. Confirmation of this thesis can be found in the work of Tiedman and Ulger [47], who stated that warming may enhance the infection window for this pathogen, in both the mid (2001–2030) and longer term (2071–2100), possibly leading to earlier infections in the future.

Our results also provide evidence for the effect of climate warming on the severity of Sclerotinia stem rot. This phenomenon is more dependent on the RCP scenario, while the influence of the locality appears to be smaller. Under the RCP4.5 scenario, nearly 60% of the simulations performed for 16 localities in four periods showed a reduction in disease severity in comparison to those registered for 1986–2005, while under RCP 8.5, this reduction was generated for nearly 90% of cases. These results are in agreement with the outcomes of Racca et al. [45], who, based on simulations, predicted a significant decrease in *Sclerotinia* infection risk, especially for the years of 2071–2100, for Lower Saxony in Germany.

In this study, we also presented the effect of the RCP scenario on the clustering of regions according to Sclerotinia stem rot severity. Under the RCP8.5 scenario, the clustering results appear to be more variable than under RCP4.5. However, under both scenarios, the lowest disease severity for all periods was generated for Zachodniopomorskie and Pomorskie. The results obtained indicate the need to adapt the protection of oilseed rape against *S. sclerotiorum* to regional conditions, reflecting the projected Sclerotinia stem rot severity for the region and period.

The future reduction in Sclerotinia stem rot severity presented in our study on the basis of the simulation outcomes results from the indirect impact of climate change. A comparison of the indirect impact and direct impact showed the superiority of the effect of the former over the latter on the Sclerotinia stem rot severity caused by climate warming in the future. Under the RCP4.5 scenario, the accumulated rate of change (AROC) for the indirect impact was greater than the AROC for the direct impact for 10 regions, while under RCP8.5, this relationship was registered for 16 regions. 

The results obtained indicated the meaning of the indirect impact of climate change on the future threat to crops of pathogens and the necessity of taking this parameter into account when predicting outbreaks of plant diseases. 

The complexity of climate change’s effects on future pathogen risk has also been highlighted by others. For example, Madgwick et al. [48] analyzed the impact of climate change on Fusarium ear blight in the UK and proved the significance of incorporating a model of host plant development into the assessment of the pathogen risk. According to these authors, the date of wheat anthesis, i.e., the growth stage at which wheat is the most vulnerable to infection with Fusarium ear blight, is expected to appear earlier by about 11–15 days across the whole country in the 2020s and 2050s. Also, Evans et al. [49,50] and Butterworth et al. [51] demonstrated that the projection of crop epidemics triggered by climate change requires both crop and pathogen development to be taken into account. The present study demonstrates an extension of this approach and numerically expresses the relationships between the direct impact and indirect impact of climate change, which covers a knowledge gap in this field. Our results highlight the role of the indirect impact in shaping disease severity and indicate that, besides the direct impact, it should be incorporated into assessment methods of climate change effects. This approach enhances our ability to project the ongoing changes driven by global warming. We propose that the accelerated development of oilseed rape in the future, caused by climate change, will contribute to the mitigation of Sclerotinia stem rot. However, we are aware that the results presented in this work cannot be generalized, because they concern only one disease and one country. Further research involving further pathogens and climatic zones is required to establish the nature of the relationship between the direct impact and indirect impact of climate change. Focusing on this problem will help to answer the question of how agriculture can adapt to or mitigate ongoing climatic changes. In our earlier study, which focused on measuring the direct impact and indirect impact of climate change on the fatty acid composition in rapeseed oil, we analyzed the relationship between these phenomena on a national scale [52]. This time, we described the relationship between the direct impact and indirect impact on a regional scale, which enabled us to present the problem more precisely.

In conclusion, it must be stressed that there are several uncertainties that impede the correct long-term projection of plant disease epidemics. Firstly, the development of the disease is influenced by many factors, among which, apart from the temperature, precipitation and humidity play very important roles. It must be pointed out that estimations of the rainfall distribution in a long time period are extremely difficult, if not impossible [37]. It is also not easy to assess long-term breeding progress; hence, there is a lack of knowledge about the phenological development of future cultivars, and their resistance to pathogens makes it difficult to predict disease severity over a period of 100 years. Nevertheless, according to Wiens et al. [53], one should not worry too much about projection uncertainties, as the alternative would be to ignore future risks. Therefore, it is believed that crop disease risk simulations, although not perfect, are helpful for the development of strategies to mitigate the effect of climate change [37].

## 5. Conclusions

The obtained results indicate the need to take the indirect impact of climate change into account when estimating the threat posed by *S. sclerotiorum* to oilseed rape in the future. This approach is worth recommending when assessing the threat posed by other pathogens to other crops. The faster development of oilseed rape expected in the future is going to mitigate the occurrence of Sclerotinia stem rot. A question arises about the reaction of other pathogens to the accelerated development of other crops driven by climate warming. Moreover, the regional differences in the threat posed by *S. sclerotiorum* to oilseed rape shown in this study indicate the need to adapt the protection of oilseed rape against this pathogen to regional conditions, reflecting the predicted severity of Sclerotinia stem rot for the region and period.

## Figures and Tables

**Table 1:** Cultivars used in the experiments aimed at model development.

Year	Cultivars		
2005	Bazyl	Kama	Kaszub
2006	Kana	Kaszub	Lisek
2007	Bazyl	Kaszub	Lisek
2008	Bazyl	Californium	Lisek
2009	Bazyl	Californium	Lisek
2010	Bazyl	Californium	Elektra
2011	Bazyl	Californium	Lisek
2012	Californium	Nelson	Visbi
2013	Artoga	Starter	Visby
2014	Artoga	Starter	Visby
2015	Artoga	Bogart	Poznaniak
2016	Bogart	Kaszub	Poznaniak
2017	Konkret	Pamela	Secure
2018	Bogart	Konkret	Pamela
2019	Anderson	Bogart	Graf
2020	Harry	Chrobry	Anderson
2021	Architekt	Graf	Harry
2022	Architekt	Graf	Harry

**Table 2:** Submodels used for the simulation of the oilseed rape flowering period.

Submodel	Formula
Gompertz + exponential	BBCH = a1 × (exp(−a2 × exp(−a3 × ARDR))) + exp(−a4 × ARDR)
Linear	BBCH = a1 + a2 × ARDR

BBCH = calculated BBCH phenological stage. ARDR = accumulated rapeseed developmental rate from sowing to the end of oilseed rape flowering. a1 to a4 = equation coefficients.

**Table 3:** Submodels used for the disease severity simulation.

Submodel	Formula
Double logistic	DS = a1 + (1/(1 + exp(a2 − a3 × T))) + a4 + (1/(1 + exp(a5 − a6 × T)))
Beta function	DS = DSopt × ((T − Tmin)/(Topt − Tmin))^n×(Topt−Tmin)/(26−Topt)^ × ((26 − T)/(26 − Topt))^n^

DS = disease severity. a1 to a6, n = equation coefficients. T = temperature. DSopt = optimal disease severity. Tmin = minimum temperature for infection. Topt = optimum temperature for infection.

**Table 4:** List of climate models used in the study.

Model	Model Source
access1-0	Centre for Australian Weather and Climate Research, Australia
access1-3	Centre for Australian Weather and Climate Research, Australia
bcc-csm1-1-m	Beijing Climate Center, China
bnu-esm	Beijing Normal University, China
canesm2	Canadian Centre for Climate Modelling and Analysis, Canada
cesm1-bgc	National Science Foundation, US Department of Energy, and National Center for Atmospheric Research, USA, and National Center for Atmospheric Research
cesm1-cam5	National Center for Atmospheric Research, USA
cmcc-cesm	Euro-Mediterranean Center on Climate Change, Italia
cmcc-cm	Euro-Mediterranean Center on Climate Change, Italia
cmcc-cms	Euro-Mediterranean Center on Climate Change, Italia
cnrm-cm5	Centre National de Recherches Météorologiques and Centre Européen de Recherche et de Formation Avancée, France
csiro-mk3-6-0	Commonwealth Scientific and Industrial Research Organization/Queensland Climate Change Centre of Excellence, Australia
gfdl-cm3	Geophysical Fluid Dynamics Laboratory, USA
gfdl-esm2g	Geophysical Fluid Dynamics Laboratory, USA
gfdl-esm2m	Geophysical Fluid Dynamics Laboratory, USA
hadgem2-ao	Met Office Hadley Centre, UK
hadgem2-es	Met Office Hadley Centre, UK
inmcm4	Institute of Numerical Mathematics, Russia
ipsl-cm5a-lr	Institut Pierre-Simon Laplace, France
ipsl-cm5a-mr	Institut Pierre-Simon Laplace, France
ipsl-cm5b-lr	Institut Pierre-Simon Laplace, France
miroc5	Atmosphere and Ocean Research Institute (The University of Tokyo), National Institute for Environmental Studies, and Japan Agency for Marine-Earth Science and Technology, Japan
miroc-esm	Japan Agency for Marine-Earth Science and Technology, Atmosphere and Ocean Research Institute (The University of Tokyo), and National Institute for Environmental Studies, Japan
miroc-esm-chem	Japan Agency for Marine-Earth Science and Technology, Atmosphere and Ocean Research Institute (The University of Tokyo), and National Institute for Environmental Studies, Japan
mpi-esm-lr	Max Planck Institute for Meteorology, Germany
mpi-esm-mr	Max Planck Institute for Meteorology, Germany
mri-cgcm3	Meteorological Research Institute, Japan
mri-esm1	Meteorological Research Institute, Japan
noresm1-m	Norwegian Climate Center, Norway

**Table 5:** Parameters of the submodels used for predicting the oilseed rape flowering period.

Submodel	Value of Coefficients in the Model					Statistical Values			
						*p*-Values			R^2^
	a1	a2	a3	a4	a1	a2	a3	a4	
Gompertz + exponential	72.4077	2.2284	0.0811	0.0338	0.0000	0.0000	0.0000	0.0000	0.92
Linear	60.9541	0.4695			0.0000	0.0000			0.81

a1 to a4 = equation coefficients.

**Table 6:** Dates of the beginning and the end of the flowering period of oilseed rape cultivars.

Year	Cultivar	Beginning of the Flowering Period			End of the Flowering Period		
		Observed	Simulated	Difference	Observed	Simulated	Difference
2006	Kana	06.05	14.05	−8	29.05	05.06	−7
	Kaszub	06.05	14.05	−8	29.05	05.06	−7
	Lisek	06.05	14.05	−8	29.05	05.06	−7
2008	Bazyl	30.04	01.05	−1	16.05	22.05	−6
	Californium	30.04	06.05	−6	16.05	22.05	−6
	Lisek	30.04	01.05	−1	16.05	22.05	−6
2010	Bazyl	30.04	09.05	−9	25.05	01.06	−7
	Californium	30.04	09.05	−9	29.05	01.06	−3
	Elektra	30.04	09.05	−9	25.05	01.06	−7
2012	Californium	07.05	01.05	6	28.05	22.05	6
	Nelson	07.05	01.05	6	28.05	22.05	6
	Visbi	07.05	01.05	6	28.05	22.05	6
2014	Artoga	21.04	16.04	5	08.05	09.05	−1
	Starter	21.04	16.04	5	08.05	09.05	−1
	Visby	21.04	16.04	5	08.05	09.05	−1
2016	Bogart	26.04	28.04	−2	17.05	20.05	−3
	Kaszub	26.04	28.04	−2	17.05	20.05	−3
	Poznaniak	26.04	28.04	−2	17.05	20.05	−3
2018	Bogart	26.04	29.04	−3	15.05	17.05	−2
	Konkret	26.04	01.05	−5	15.05	22.05	−7
	Pamela	26.04	29.04	−3	15.05	17.05	−2
2020	Harry	24.04	20.04	4	17.05	15.05	2
	Chrobry	24.04	20.04	4	17.05	15.05	2
	Anderson	24.04	20.04	4	17.05	15.05	2
2022	Architekt	29.04	30.04	−1	19.05	20.05	−1
	Elevation	29.04	30.04	−1	19.05	20.05	−1
	Harry	29.04	30.04	−1	19.05	20.05	−1

**Table 7:** Parameters of the submodels used for estimating sclerotinia stem rot severity in oilseed rape at flowering time.

Model	Values of Coefficients in the Model						Statistical Values						
							*p*-Values						R^2^
Double logistic	a1	a2	a3	a4	a5	a6	a1	a2	a3	a4	a5	a6	0.99
	0.807	11.49	0.737	−1.70	3.734	0.976	0.000	0.000	0.000	0.000	0.000	0.000	
Beta function	DSopt	Tmin	Topt	n			DSopt	Tmin	Topt	n			0.98
	0.979	12.78	19.49	1.099			0.007	0.000	0.000	0.016			

a1 to a6, DSopt, Tmin, Topt, n = equation coefficients.

**Table 8:** The reduction in time needed for the oilseed rape to start (BBCH 60) and end (BBCH 69) flowering and the duration of the flowering period predicted for the two scenarios (RCP4.5, RCP8.5) and four periods (2020–2039, 2040–2059, 2060–2079, 2080–2099) in comparison to those registered in the period of 1986–2005.

Region	BBCH 60									BBCH 69									Flowering Period (Days)								
	1986–2005	RCP4.5				RCP8.5				1986–2005	RCP4.5				RCP8.5				1986–2005	RCP4.5				RCP8.5			
		2020–2039	2040–2059	2060–2079	2080–2099	2020–2039	2040–2059	2060–2079	2080–2099		2020–2039	2040–2059	2060–2079	2080–2099	2020–2039	2040–2059	2060–2079	2080–2099		2020–2039	2040–2059	2060–2079	2080–2099	2020–2039	2040–2059	2060–2079	2080–2099
Dolnośląskie	124	10	14	18	20	11	20	30	38	145	9	13	16	18	9	17	25	34	21	22	22	23	23	23	24	26	25
Kujawsko-Pomorskie	128	10	14	17	20	10	19	29	38	149	10	14	17	19	10	18	27	35	21	21	21	21	22	21	22	23	24
Lubelskie	129	8	11	15	17	8	16	25	33	149	8	12	15	16	8	15	23	30	20	20	19	20	21	20	21	22	23
Lubuskie	122	12	17	21	24	13	23	34	44	143	11	15	19	21	11	20	30	39	21	22	23	23	24	23	24	25	26
Łódzkie	126	9	12	16	18	9	17	26	35	146	8	11	15	16	9	15	23	31	20	21	21	21	22	20	22	23	24
Małopolskie	136	9	13	17	18	10	17	26	35	157	9	13	16	18	9	16	25	33	21	21	21	22	21	22	22	22	23
Mazowieckie	128	10	14	17	20	11	19	29	38	148	10	13	17	19	10	17	26	34	20	20	21	20	21	21	22	23	24
Opolskie	123	10	14	18	20	10	19	29	38	144	9	13	17	19	10	17	26	34	21	22	22	22	22	21	23	24	25
Podkarpackie	131	9	13	16	18	10	17	26	35	152	10	13	17	18	10	17	25	33	21	20	21	20	21	21	21	22	23
Podlaskie	135	10	14	17	19	10	18	28	38	155	10	14	17	19	10	17	26	34	20	20	20	20	20	20	21	22	24
Pomorskie	135	13	17	22	25	13	23	35	46	157	12	16	21	23	12	21	32	41	22	23	23	23	24	23	24	25	27
Śląskie	127	9	13	16	18	9	18	27	36	148	9	13	16	18	9	16	25	33	21	21	21	21	21	21	23	23	24
Świętokrzyskie	128	9	12	16	18	9	17	27	36	148	9	12	16	17	9	16	24	32	20	20	20	20	21	20	21	23	24
Warmińsko-Mazurskie	133	10	14	18	20	10	19	29	39	154	10	14	18	20	10	18	27	36	21	21	21	21	21	21	22	23	24
Wielkopolskie	123	10	14	18	20	10	19	29	38	144	10	13	17	19	10	17	26	34	21	21	22	22	22	21	23	24	25
Zachodniopomorskie	128	12	17	21	24	13	23	34	44	150	11	15	19	21	12	20	30	39	22	23	24	24	25	23	25	26	27
Minimum	122	8	11	15	17	8	16	25	33	143	8	11	15	16	8	15	23	30	20	20	19	20	20	20	21	22	23
Maximum	136	13	17	22	25	13	23	35	46	157	12	16	21	23	12	21	32	41	22	23	24	24	25	23	25	26	27

**Table 9:** The Sclerotinia stem rot severity simulated for sixteen regions under two scenarios (RCP4.5, RCP8.5) and four periods (2020–2039, 2040–2059, 2060–2079, 2080–2099) in comparison to those registered in the period of 1986–2005.

Region	1986–2005	RCP4.5				RCP8.5			
		2020–2039	2040–2059	2060–2079	2080–2099	2020–2039	2040–2059	2060–2079	2080–2099
Dolnośląskie	8.36	8.19	8.23	8.09	7.87	8.11	7.63	7.06	6.54
Kujawsko-Pomorskie	8.84	8.73	8.74	8.73	8.51	8.73	8.25	7.71	7.05
Lubelskie	9.14	9.28	9.35	9.51	9.44	9.23	9.05	8.96	8.42
Lubuskie	8.14	7.99	7.71	7.40	7.21	7.92	7.04	6.34	5.96
Łódzkie	9.00	8.85	8.96	9.01	8.80	8.82	8.54	8.25	7.65
Małopolskie	8.16	8.21	8.17	8.26	8.27	8.17	7.87	8.16	8.02
Mazowieckie	8.99	9.04	9.10	9.18	9.00	9.01	8.69	8.22	7.56
Opolskie	8.08	8.16	8.22	8.17	7.99	8.05	7.68	7.28	6.81
Podkarpackie	8.21	8.27	8.34	8.57	8.60	8.25	8.13	8.40	8.12
Podlaskie	9.16	9.01	9.03	9.18	9.06	9.05	8.72	8.50	8.08
Pomorskie	7.10	7.00	6.90	6.73	6.53	7.01	6.40	5.84	5.51
Śląskie	8.85	8.70	8.81	8.93	8.84	8.67	8.44	8.34	7.69
Świętokrzyskie	8.95	8.95	9.07	9.24	9.14	8.96	8.70	8.60	7.94
Warmińsko-Mazurskie	8.78	8.49	8.53	8.53	8.39	8.51	8.14	7.81	7.38
Wielkopolskie	8.92	8.70	8.63	8.42	8.20	8.62	7.95	7.21	6.64
Zachodniopomorskie	7.04	6.97	6.93	6.72	6.52	6.93	6.39	5.78	5.40
Coefficient of variation	0.076	0.077	0.084	0.097	0.103	0.078	0.097	0.124	0.128

**Table 10:** Direct and indirect impacts of climate change generated for the two scenarios (RCP4.5, RCP8.5) and four periods (2020–2039, 2040–2059, 2060–2079, 2080–2099).

Region	Direct Impact								Indirect Impact							
	RCP4.5				RCP8.5				RCP4.5				RCP8.5			
	2020–2039	2040–2059	2060–2079	2080–2099	2020–2039	2040–2059	2060–2079	2080–2099	2020–2039	2040–2059	2060–2079	2080–2099	2020–2039	2040–2059	2060–2079	2080–2099
Dolnośląskie	1.87	2.69	3.36	3.50	1.93	2.78	4.36	5.55	−2.03	−2.83	−3.63	−3.99	−2.19	−3.52	−5.66	−7.38
Kujawsko-Pomorskie	1.66	2.32	2.93	3.10	1.76	2.52	3.79	4.73	−1.76	−2.42	−3.04	−3.43	−1.88	−3.11	−4.92	−6.51
Lubelskie	1.75	2.39	3.06	3.24	1.83	2.59	3.90	4.78	−1.60	−2.17	−2.68	−2.94	−1.74	−2.67	−4.08	−5.50
Lubuskie	1.85	2.47	3.27	3.57	1.90	2.85	4.46	5.62	−2.00	−2.90	−4.01	−4.50	−2.11	−3.95	−6.26	−7.80
Łódzkie	1.62	2.34	2.88	2.93	1.72	2.44	3.73	4.64	−1.78	−2.38	−2.87	−3.14	−1.91	−2.90	−4.48	−5.99
Małopolskie	2.32	3.29	4.18	4.46	2.28	3.50	5.39	6.63	−2.27	−3.28	−4.08	−4.35	−2.27	−3.80	−5.38	−6.77
Mazowieckie	1.73	2.33	3.00	3.21	1.81	2.59	3.88	4.78	−1.68	−2.22	−2.81	−3.20	−1.80	−2.89	−4.65	−6.22
Opolskie	1.95	2.75	3.52	3.74	1.99	2.91	4.64	5.97	−1.87	−2.61	−3.43	−3.84	−2.02	−3.31	−5.45	−7.25
Podkarpackie	2.12	2.88	3.78	4.10	2.12	3.21	5.06	6.26	−2.05	−2.74	−3.42	−3.70	−2.07	−3.29	−4.87	−6.34
Podlaskie	1.87	2.59	3.29	3.51	2.00	2.89	4.15	4.97	−2.02	−2.72	−3.27	−3.62	−2.11	−3.32	−4.81	−6.04
Pomorskie	1.98	3.01	3.88	4.13	2.12	3.45	5.38	7.06	−2.07	−3.21	−4.25	−4.70	−2.21	−4.15	−6.64	−8.65
Śląskie	1.92	2.65	3.36	3.57	1.93	2.79	4.31	5.32	−2.07	−2.69	−3.28	−3.58	−2.11	−3.20	−4.83	−6.48
Świętokrzyskie	1.91	2.57	3.32	3.56	1.92	2.78	4.29	5.28	−1.91	−2.45	−3.03	−3.37	−1.91	−3.02	−4.64	−6.29
Warmińsko-Mazurskie	1.67	2.40	3.03	3.25	1.80	2.67	3.89	4.80	−1.96	−2.65	−3.28	−3.64	−2.07	−3.31	−4.86	−6.21
Wielkopolskie	1.69	2.35	2.94	3.06	1.77	2.48	3.78	4.71	−1.90	−2.63	−3.43	−3.78	−2.07	−3.45	−5.49	−6.99
Zachodniopomorskie	1.66	2.51	3.21	3.30	1.78	2.79	4.51	6.08	−1.73	−2.62	−3.53	−3.82	−1.89	−3.44	−5.76	−7.71

## Data Availability

The data supporting the results of this study are available from the authors (M.W. or A.W.) upon reasonable request.
